# Out-of-hospital cardiac arrest outcomes’ determinants: an Italian retrospective cohort study based on Lombardia CARe

**DOI:** 10.1007/s11739-024-03573-z

**Published:** 2024-03-28

**Authors:** Alice Clara Sgueglia, Leandro Gentile, Paola Bertuccio, Maddalena Gaeta, Margherita Zeduri, Daniela Girardi, Roberto Primi, Alessia Currao, Sara Bendotti, Gianluca Marconi, Giuseppe Maria Sechi, Simone Savastano, Anna Odone

**Affiliations:** 1 Department of Public Health, Experimental and Forensic Medicine, University of Pavia, Via Forlanini 2, 27100 Pavia, Italy; 2 Division of Cardiology, Fondazione IRCCS Policlinico San Matteo, Pavia, Italy; 3 Agenzia Regionale Emergenza Urgenza AREU Lombardia, Milan, Italy; 4 Medical Direction, Fondazione IRCCS Policlinico San Matteo, Pavia, Italy

**Keywords:** Out-of-hospital cardiac arrest, Hospital mortality, Delivery of Health Care, Emergency medical services, Resource allocation

## Abstract

This study on the Lombardia Cardiac Arrest Registry (Lombardia CARe,) the most complete nationwide out-of-hospital cardiac arrest (OHCA) registry in Italy, aims at evaluating post-OHCA intra-hospital mortality risk according to patient’s characteristics and emergency health service management (EMS), including level of care of first-admission hospital. Out of 12,581 patients included from 2015 to 2022, we considered 1382 OHCA patients admitted alive to hospital and survived more than 24 h. We estimated risk ratios (RRs) of intra-hospital mortality through log-binomial regression models adjusted by patients’ and EMS characteristics. The study population consisted mainly of males (66.6%) most aged 60–69 years (24.7%) and 70–79 years (23.7%). Presenting rhythm was non-shockable in 49.9% of patients, EMS intervention time was less than 10 min for 30.3% of patients, and cardiopulmonary resuscitation (CPR) was performed for less than 15 min in 29.9%. Moreover, 61.6% of subjects (n = 852) died during hospital admission. Intra-hospital mortality is associated with non-shockable presenting rhythm (RR 1.27, 95% CI 1.19–1.35) and longer CPR time (RR 1.39, 95% CI 1.28–1.52 for 45 min or more). Patients who accessed to a secondary vs tertiary care hospital were more frequently older, with a non-shockable presenting rhythm and longer EMS intervention time. Non-shockable presenting rhythm accounts for 27% increased risk of intra-hospital death in OHCA patients, independently of first-access hospital level, thus demonstrating that patients’ outcomes depend only by intrinsic OHCA characteristics and Health System’s resources are utilised as efficiently as possible.

## Introduction

Out-of-hospital cardiac arrests (OHCAs)—defined as the cessation of cardiac mechanical activity and the absence of signs of systemic circulation, occurring outside a hospital setting [1]—are associated with low survival rates, poor neurological outcomes and are responsible for severe social burden at the global level [2–4].

It is estimated that OHCA has a global incidence among adults of 55/100,000 [5], with 300,000 cases occurring in Europe every year [6]. OHCA is the most time-critical and time-dependent medical emergency [7], with survival strictly relying on a strong “chain of survival” [8] that involves integrated and early interventions: recognition of cardiac arrest, bystanders cardiopulmonary resuscitation (CPR), defibrillation, advanced hospital care and appropriate post resuscitation treatments [9, 10]. Many pre-hospital factors influence OHCA outcomes, including the place where the event occurs, bystander CPR, initial cardiac rhythm, early defibrillation and transport time interval (TTI) [11, 12]. Among these factors, the Emergency Medical Service response (EMS) time has a considerable effect on OHCA outcomes, [12] with short EMS response time associated with higher rates of survival to hospital discharge and favourable neurological outcomes after the event [3, 11, 13, 14]. OHCA survival strongly correlates with early CPR performed by bystanders or healthcare professionals with automated external defibrillator (AED) in shockable rhythms [15, 16] with survival rate decreasing for every minute defibrillation is delayed [17].

The identification of best OHCA management models remains controversial and they vary widely by country and health services organisation [18]. The majority of patients who experience OHCA are often transported to the closest emergency department (ED) [19, 20], and some are subsequently transferred for specialty care [21, 22]; higher survival rates and better outcomes are associated with transport to a specialised cardiac arrest centre or critical care medical centre [23, 24]. However, despite this evidence, there are still gaps in knowledge on determinants of survival among OHCA patients, also because of the huge variations of EMS networks and treatments in different regions of the world [25]. To quantify and monitor OHCA burden, as well as to assess and evaluate OHCA management models and performances, several countries in the last decades have implemented and maintained registries [26], demonstrating the key role of using real data from different settings in tailoring optimal OHCA management models.

In Italy, where an average of 60,000 OHCAs occur per year, with an incidence of 1 per 1000 inhabitants [27], data from the Cardiac Arrest Registry of the Lombardy region (Lombardia CARe) [28] provides timely and comprehensive data on OHCA burden. Based on this registry, this study aimed at evaluating post-OHCA intra-hospital mortality risk in relation to patient’s characteristics and emergency health service management, including level of care of first admission hospital.

## Methods

### Study design and setting

We conducted a retrospective cohort study, based on data collected from the Cardiac Arrest Registry of the Lombardy region (Lombardia CARe; clinical trial identifier on clinicaltrials.gov NCT03197142, approval reference 20140028219 by the Ethical Committee of Fondazione IRCCS Policlinico San Matteo in Pavia), started in 2015 [27]. This multicentre longitudinal prospective registry is the most complete nationwide, gathering automated data on OHCAs occurring in Lombardy region, collected in compliance with 2014 Utstein criteria [29]. Lombardia CARe covers a total area of 15,126 km^2^ divided into seven provinces: Brescia, Como, Cremona, Lodi, Mantova, Pavia and Varese. Each province features several rural regions and a few urban areas for a total population of more than 4 million inhabitants; population density varies depending on the territorial characteristics and the level of urbanisation. The road network differs ranging from fast-flowing motorways to narrow and crooked hillside roads. In the whole Region emergency medical services are coordinated and provided by “Agenzia Regionale Emergenza Urgenza” (AREU), that ensures the standardisation of medical procedures in all covered areas. Information regarding every intervention is gathered and archived in an electronic database connected to Lombardia CARe.

### Data management

Data were collected and managed using REDCap (Research Electronic Data Capture) tools hosted at Fondazione IRCCS Policlinico San Matteo [30]. REDCap is a web-based application designed to support data capture for research studies, providing: an intuitive interface for validated data entry, audit trails for tracking data manipulation and export procedures, automated export procedures for data downloads to common statistical packages, and procedures for data import from external sources. Pre-hospital information of each OHCA is automatically gathered from the AREU data warehouse, filled in a database and subsequently checked and validated by local EMS personnel. In each hospital a team of specialists manages clinical information regarding both the in-hospital stay and the outcomes. Pre-hospital, in-hospital and follow-up information is archived in Lombardia CARe and data quality is assured by a dedicated study management team.

### Study population and variables of interest

Between the 1st of January 2015 and the 31st of December 2022, 12,581 OHCAs were recorded in Lombardia CARe within the provinces of Brescia, Como, Cremona, Lodi, Mantova, Pavia and Varese. Among them, we considered patients who: suffered from OHCA by medical aetiology, were admitted alive to hospital and survived more than 24 h after hospital admission. Informed consent forms were collected from patients who survived up to hospital discharge with a good neurological outcome; subsequently personal information was anonymised.

Our primary outcome of interest was post-OHCA intra-hospital mortality. We considered the following exposure variables: selected patients’ characteristics (sex, age and presenting rhythm), selected EMS parameters (use of public access AED, intervention time and CPR duration) and the first access hospital’s level of care (secondary or tertiary).

Concerning hospital’s level of care, we considered as secondary level the hospitals differentiated by function and with 5–10 clinical specialities, usually identified as provincial (spoke). On the other hand, tertiary level was assigned to highly specialised referral hospitals, with clinical services extremely differentiated by function and occasionally linked to Universities (hub). Tertiary care hospitals may also host a cardiac arrest centre, a high-volume or regionalised institution treating OHCA patients with post resuscitation care, including 24/7 access to a cardiac catheterisation laboratory for coronary angiography and percutaneous coronary intervention [31].

### Statistical analysis

We estimated the risk ratio (RR) of intra-hospital mortality and corresponding 95% confidence intervals (CI) through log-binomial regression models adjusted by variables resulted statistically significant in the univariate models. We performed two separate models, one (Model 1) partially adjusted by sex and age group (years 0–49, 50–59, 60–69, 70–79, ≥ 80), and one (Model 2) further adjusted by presenting rhythm (shockable or non-shockable), CPR duration (minutes < 15, 15 ≤ 30, 30 ≤ 45, ≥ 45) and level of hospital care at first admission (secondary or tertiary). In addition, to investigate associations between hospital level and selected characteristics, we used the Chi-square test and estimated odds ratios (OR) and relative 95% CI through adjusted logistic regression models. The latter models were mutually adjusted by sex, age group (years 0–49, 50–59, 60–69, 70–79, ≥ 80), presenting rhythm (shockable or non-shockable), EMS intervention time (minutes < 10, 10 ≤ 14, ≥ 14, according to the tertiles of our data distribution).

## Results

1382 patients from Lombardia CARe met our inclusion criteria and were included in the study population. Table 1 reports the distribution of patients, overall and by study outcome, according to province and selected characteristics. Study population consisted mainly of males (66.6%) and belonging to the age groups 60–69 years (24.7%) and 70–79 years (23.7%). Most OHCAs occurred at home (74.3%). First CPR was performed by bystanders for 38.5% of patients, and by other (i.e. EMS operators or first responders) for 46.8% of them; telephone CPR was carried out for 34.5% of the patients. Presenting rhythm was non-shockable in 49.9% of patients and the public access defibrillation was used for a minor part of the population in study (4.7%). For 30.3% of patients the EMS took no longer than 10 min to arrive and on 29.9% of patients the CPR was performed for less than 15 min. VA-ECMO was used in 42 patients, with an average age of 53 years. Overall, 43.1% of patients were admitted to a secondary care hospital, and 56.9% to a tertiary care one. 61.6% of subjects (n = 852) died during hospital admission. The percentage of patients who died was higher among females (69.1%), older patients (73.7% at age 80–89, and 77.2% at age 90 or over), among those who had a non-shockable presenting rhythm (83.3%) and for patients on whom a public access AED was not used (62.5%). Moreover, a higher percentage of deaths was observed at increasing CPR duration and for those who were admitted to secondary care hospitals (67.7%).

**Table 1:** Distribution of 1382 patients, overall and by study outcome, according to province and selected characteristics

	Overall		Alive		Dead		*p *value*
	n	%	n	%	n	%	
Overall	1382		530	38.4	852	61.6	
Province							0.01
Brescia	142	10.3	55	38.7	87	61.3	
Como	85	6.2	32	37.6	53	62.4	
Cremona	158	11.4	49	31.0	109	69.0	
Lodi	92	6.7	31	33.7	61	66.3	
Mantova	130	9.4	54	41.5	76	58.5	
Pavia	535	38.7	234	43.7	301	56.3	
Varese	240	17.4	75	31.2	165	68.8	
Sex							< 0.01
Male	921	66.6	388	42.1	533	57.9	
Female	460	33.3	142	30.9	318	69.1	
Missing	1	0.1	0	–	1	–	
Age group (years)							< 0.01
0–9	6	0.4	1	16.7	5	83.3	
10–19	3	0.2	1	33.3	2	66.7	
20–29	11	0.8	6	54.5	5	45.5	
30–39	27	2.0	13	48.1	14	51.9	
40–49	104	7.5	52	50	52	50.0	
50–59	243	17.6	115	47.3	128	52.7	
60–69	341	24.7	144	42.2	197	57.8	
70–79	328	23.7	116	35.4	212	64.6	
80–89	259	18.7	68	26.3	191	73.7	
90 +	57	4.1	13	22.8	44	77.2	
Missing	3	0.2	1	–	2	–	
OHCA location							NS
Home	1027	74.3	378	36.8	649	63.2	
Other	325	23.5	138	42.5	187	57.5	
Missing	30	2.2	14	–	16	–	
Bystanders CPR							NS
Yes	532	38.5	197	37.0	335	63.0	
Other^a^	647	46.8	234	36.2	413	63.8	
Missing	203	14.7	99	–	104	–	
Telephone CPR							NS
No	890	64.4	349	39.2	541	60.8	
Yes	477	34.5	175	36.7	302	63.3	
Missing	15	1.1	6	–	9	–	
Presenting rhythm							< 0.01
Shockable	675	48.8	407	60.3	268	39.7	
Non-schockable	690	49.9	115	16.7	575	83.3	
Missing	17	1.2	8	–	9	–	
Public Access AED use							< 0.01
No	1316	95.2	493	37.5	823	62.5	
Yes	65	4.7	37	56.9	28	43.1	
Missing	1	0.1	0	–	1	–	
EMS intervention time (minutes)							NS
< 10	419	30.3	167	39.9	252	60.1	
10 ≤ 14	484	35.0	194	40.1	290	59.9	
≥ 14	474	34.3	167	35.2	307	64.8	
Missing	5	0.4	2	–	3	–	
CPR duration (minutes)							< 0.01
< 15	413	29.9	276	66.8	137	33.2	
15 ≤ 30	412	29.8	173	42.0	239	58.0	
30 ≤ 45	256	18.5	59	23.0	197	77.0	
≥ 45	301	21.8	22	7.3	279	92.7	
VA-ECMO use							< 0.01
No	1323	95.7	530	40.1	793	59.9	
Yes	42	3.1	0	0.0	42	100.0	
Missing	17	1.2	–	–	17	–	
Hospital level							< 0.01
Secondary care hospital	595	43.1	192	32.3	403	67.7	
Tertiary care hospital	787	56.9	338	42.9	449	57.1	

Lombardia CARe, Italy, 2015–2022

*AED* automated external defibrillators, *CPR* cardiopulmonary resuscitation, *EMS* emergency medical services, *NS* not significant, *VA-ECMO* veno-arterial extracorporeal membrane oxygenation

*P value from the Chi-square test

^a^Performed by EMS or first responder

Table 2 reports results from the unadjusted and adjusted log-binomial regression models in terms of RR of intra-hospital mortality and corresponding 95% confidence intervals (CI). According to Model 1 (i.e., mutually adjusted by sex and age), mortality risk was higher in females than males (RR 1.11, 95% CI 1.02–1.21), in older patients (RR 1.24, 95% CI 1.04–1.47 at age 70–79 and RR 1.38, 95% CI 1.17–1.64 at age 80 or over) with a significant *p *value for trend at increasing age; in patients presenting a non-shockable rhythm compared to shockable rhythm (RR 2.07, 95% CI 1.87–2.29). Compared to patients on whom CPR was performed less than 15 min, the risk increased for longer times (*p* for trend < 0.01). Conversely, the public access AED use was associated with a reduced intra-hospital mortality risk (RR 0.74, 95% CI 0.56–0.98), as well as the admission to a tertiary vs. secondary care hospital (RR 0.87, 95% CI 0.81–0.95). When considering Model 2 (i.e., mutually adjusted by age, sex, presenting rhythm, CPR time and hospital level), statistically significant associations with increased intra-hospital mortality risk remained for non-shockable rhythm (RR 1.27, 95% CI 1.19–1.35), and at increasing of CPR time (RR 1.39, 95% CI 1.28–1.52 for 45 min or more; *p *value for trend < 0.01). No association emerged between intra-hospital mortality risk and the level of hospital care at first admission.

**Table 2:** Results from the unadjusted and adjusted log-binomial regression models in terms of risk ratios (RR) of intra-hospital mortality and corresponding 95% confidence intervals (CI) among the 1382 patients

	Crude^a^ RR (95% CI)	Model 1: adjusted^b^ RR (95% CI)	Model 2: adjusted^c^ RR (95% CI)
Sex			
Male	1^d^	1^d^	1^d^
Female	1.19 (1.10–1.30)	1.11 (1.02–1.21)	0.99 (0.94–1.05)
Age group (years)			
0–49	1^d^	1^d^	1^d^
50–59	1.02 (0.84–1.24)	1.02 (0.84–1.24)	1.05 (0.92–1.19)
60–69	1.12 (0.94–1.34)	1.11 (0.93–1.33)	1.02 (0.92–1.13)
70–79	1.25 (1.05–1.49)	1.24 (1.04–1.47)	1.04 (0.94–1.15)
80 +	1.44 (1.22–1.70)	1.38 (1.17–1.64)	1.09 (0.98–1.21)
*P* for trend	< 0.01	< 0.01	0.24
Presenting rhythm			
Shockable	1^d^	1^d^	1^d^
Non-shockable	2.10 (1.90–2.32)	2.07 (1.87–2.29)	1.27 (1.19–1.35)
Public access AED use			
AED not used	1^d^	1^d^	1^d^
AED used	0.69 (0.52–0.91)	0.74 (0.56–0.98)	0.91 (0.76–1.09)
EMS intervention time (min)			
< 10	1^d^	1^d^	1^d^
10 ≤ 14	1.00 (0.90–1.11)	0.99 (0.90–1.10)	0.98 (0.93–1.03)
≥ 14	1.08 (0.97–1.19)	1.08 (0.98–1.19)	1.01 (0.95–1.06)
*P* for trend	0.14	0.12	0.64
CPR duration (min)			
< 15	1^d^	1^d^	1^d^
15 ≤ 30	1.75 (1.49–2.05)	1.74 (1.49–2.04)	1.20 (1.10–1.31)
30 ≤ 45	2.32 (1.99–2.70)	2.32 (1.99–2.70)	1.34 (1.23–1.46)
≥ 45	2.79 (2.43–3.22)	2.67 (2.31–3.08)	1.39 (1.28–1.52)
*P* for trend	< 0.01	< 0.01	< 0.01
Hospital level			
Secondary care hospital	1^d^	1^d^	1^d^
Tertiary care hospital	0.84 (0.78–0.91)	0.87 (0.81–0.95)	1.01 (0.96–1.06)

Lombardia CARe, 2015–2022

^a^Estimates obtained through unadjusted log-binomial regression model

^b^Estimates obtained through log-binomial regression model 1, adjusted by sex and age group (0–49, 50–59, 60–69, 70–79, ≥ 80)

^c^Estimates obtained through log-binomial regression model 2, adjusted by sex, age group (0–49, 50–59, 60–69, 70–79, ≥ 80), presenting rhythm (shockable or non-shockable), CPR duration (< 15, 15 ≤ 30, 30 ≤ 45, 45–90), hospital level (secondary or tertiary)

^d^Reference category

Table 3 reports the distribution of OHCA patients at admission by hospital level according to selected characteristics, along with odds ratios (OR) for hospital level and corresponding 95% confidence interval (CI). Patients who accessed a tertiary care hospital, compared to those who accessed a secondary care one, were more frequently males (70.1% vs. 62.0%, respectively), younger (mean age 65.3 vs. 68.8 years), and presented a shockable rhythm (56.0% vs. 39.3%). The EMS intervention time was more than 14 min for 30.9% of patients admitted at tertiary care hospital, compared to 38.8% of those arriving at secondary care hospital; CPR time was 45 min or more for 24.3% and 18.5%, respectively. After adjusting for selected covariates, the likelihood of accessing a tertiary care level was lower at increasing age (OR 0.48, 95% CI 0.32–0.73 at age 80 or over, *p* for trend < 0.01), for patients with a non-shockable presenting rhythm (OR 0.58, 95% CI 0.46–0.73) and at increasing EMS intervention time (OR 0.67, 95% CI 0.51–0.89 for times longer than 14 min, *p* for trend < 0.01).

**Table 3:** Distribution of 1382 patients at admission by hospital level, according to selected characteristics, along with odds ratios (OR) and corresponding 95% confidence interval (CI) (tertiary *versus* secondary care hospital)

Overall	Secondary care hospital		Tertiary care hospital		*p *value^a^	OR^b^ (95% CI)
	n	%	n	%		
	595	43.1	787	56.9		
Sex					< 0.01	
Male	369	62.0	552	70.1		1
Female	225	37.8	235	29.9		0.94 (0.73–1.20)
Missing		–	–	–		
Age group (years)					< 0.01	
0–49	52	8.7	99	12.6		1
50–59	79	13.3	164	20.8		1.06 (0.68–1.64)
60–69	137	23.0	204	25.9		0.79 (0.53–1.19)
70–79	149	25.0	179	22.7		0.70 (0.46–1.05)
80 +	176	29.6	140	17.8		0.48 (0.32–0.73)
Mean (SD)	69.8 (14.7)		65.3 (14.5)			
*P* for trend						< 0.01
Missing	2	–	1	–		
OHCA location					NS	
Home	459	77.1	568	72.2		1
Other	127	21.3	198	25.2		1.01 (0.77–1.32)
Missing	9	–	21	–		
Who performed the first CPR?					< 0.01	
Bystander	226	38.0	306	38.9		1
First responder	4	0.7	1	0.1		0.21 (0.02–1.94)
EMS operators	324	54.5	318	40.4		0.87 (0.68–1.11)
Missing	41	–	162	–		–
Telephone CPR					< 0.01	
Yes	173	29.1	304	38.6		1
No	415	69.7	475	60.4		0.81 (0.63–1.02)
Missing	7	–	8	–		
Presenting rhythm					< 0.01	
Shockable	234	39.3	441	56.0		1
Non-shockable	352	59.2	338	42.9		0.58 (0.46–0.73)
Missing	9	–	8	–		
Public access AED use					NS	
Yes	21	3.5	44	5.6		1
No	573	96.3	743	94.4		0.80 (0.46–1.38)
Missing	1	–	–	–		
EMS intervention time (minutes)					< 0.01	
< 10	160	26.9	259	32.9		1
10 ≤ 14	202	33.9	282	35.8		0.86 (0.65–1.13)
≥ 14	231	38.8	243	30.9		0.67 (0.51–0.89)
*P* for trend						< 0.01
Missing	2	–	3	–		
CPR duration (minutes)					0.03	
< 15	179	30.1	234	29.7		1
15 ≤ 30	180	30.3	232	29.5		1.07 (0.80–1.43)
30 ≤ 45	126	21.2	130	16.5		0.82 (0.59–1.14)
≥ 45	110	18.5	191	24.3		1.35 (0.98–1.86)
*P* for trend						0.25

Lombardia CARe, Italy, 2015–2022

^a^*P *value from the Chi-square test

^b^Estimates obtained through logistic regression model, adjusted by sex, age group (0–49, 50–59, 60–69, 70–79, ≥ 80), presenting rhythm (shockable or non-shockable), and EMS intervention time (< 10, 10 ≤ 14, ≥ 14)

## Discussion

Our analysis, based on a retrospective cohort of patients suffering an OHCA over the period 2015–2022, showed that non-shockable presenting rhythm was associated with a 27% increased risk of intra-hospital mortality, independently from OHCA models of care or first access type of hospital. As a further result, longer CPR duration was associated with a higher risk of death within hospital admission (20% for 15 to ≤ 30 min, up to 39% for 45 min or more).

Our study highlights that presenting rhythm is decisive in determining survival. Our sample strongly confirms that among shockable patients death rate is just under 40%, while for non-shockable patients this rate doubles, rising to over 80%. This result can be explained considering that the type of rhythm presented after an OHCA episode is directly linked to the severity of the event. Therefore, it can serve as a good proxy for its measurement, given its substantial influence on both short- and long-term treatment options. Indeed, among the possible rhythms shown by patients suffering from cardiac arrest (ventricular fibrillation, pulseless ventricular tachycardia, pulseless electrical activity and asystole), pulseless electrical activity and asystole are defined as non-shockable. Consequently, these patients tend to have worse prognoses and higher mortality rates, as shown by existing literature [32–35] and confirmed by our analysis.

Secondly, this study indicates that longer CPR durations are linked to an increase in mortality risk. This originates from the fact that long and difficult CPRs are a consequence of OHCAs characterised by greater severity. Despite the fact that a CPR duration of 20–30 min is usually considered as an appropriate cutoff point [36], some studies show that patients might benefit from longer resuscitation periods [37, 38]. However, there is solid evidence in literature pointing to the fact that survival rate decreases as CPR duration increases [39, 40].

The focus of our study was to investigate the role of level of care of first admission hospital (secondary or tertiary) on post-OHCA intra-hospital mortality risk. According to our findings, admission to a tertiary care hospital was identified as a prognostic factor for survival only when adjusted for sex and age (Model 1), while further adjusting by presenting rhythm and CPR duration (Model 2) the level of care was no longer significant. Literature shows wide variations between studies concerning the impact of hospital characteristics. Some studies suggested that a higher level of care, including cardiac arrest centres, is associated to improved survival rates [31, 41, 42], while others claimed that the level of care is not an independent predictor of survival [31, 43]. In our analysis, the fact that level of care did not show a significant effect on intra-hospital mortality risk may likely indicate that patients were properly assigned to the hospital of care according to their needs. Therefore, we can assume that the EMS management model has been optimally working in Lombardy over the past eight calendar years. Deepening into this aspect and in order to explore the decision-making process behind triage, we analysed the characteristics of patients who accessed secondary care hospitals compared to those who accessed tertiary care hospitals. Data showed that EMS operators assigned patients according to variables strongly linked to the severity of the event, including age, presenting rhythm and intervention time, even if there is no standardised protocol or pathway to guide them. The resulting management model implies that older patients with non-shockable rhythm and longer intervention time (therefore with a more severe prognosis) are assigned to secondary care hospitals (spoke). This is aimed at rationalising resources of tertiary level hospitals and cardiac arrest centres (hub) for patients with less severe OHCA and a likely better prognosis. The no role of first admission hospital’s level of care as a prognostic factor demonstrates that decision-making and risk prediction tools used by EMS are functioning effectively: they ensure that patients’ outcomes mainly reflect intrinsic OHCA characteristics and the event’s severity, and the Health System’s resources are utilised correctly. Considering the impact of first admission hospital and patient’s characteristics, literature on OHCA often takes into account the possible presence of ST-elevation at the ECG after resuscitation. Even if there are conflicting results [31], post-arrest care provided in a cardiac arrest centre has likely better outcomes [44, 45]; however a recent trial reported that in adult patients without ST-elevation, transfer to a cardiac arrest centre following resuscitated cardiac arrest in the community did not reduce mortality [46].

Our findings should be considered within the context of the study’s limitations, intrinsically linked to the data-gathering methodology of Lombardia CARe. First, the Registry does not cover the whole of Lombardy but only seven of its Provinces: subsequently, despite the high number of inhabitants, it does not provide a fully representative overview of the Region. Besides, health conditions, previous diseases and lifestyles of the patients in study remain unknown as in the vast majority of studies about OHCA. However, we were able to adjust the models not only by sex and age, but also by presenting rhythm as a proxy for the severity of the event, first admission hospital’s level of care and CPR time, which represent the most relevant confounders for the aim of this study. In addition, it has to be kept into account that Italy has been deeply affected by the COVID-19 pandemic, which peaked during the years 2020 and 2021. This extraordinary event may have affected the number of OHCAs brought to the attention of the Health System and subsequently collected within the Registry and may have impacted transport management and EMS timing. In fact, evidence reports that OHCA incidence and mortality were higher in pandemic emergency times [47], and there were significant changes in resuscitation practices during the COVID-19 pandemic [48–51]. In contrast, other studies showed no significant differences in OHCA survival and neurological prognosis compared to pre-pandemic times, possibly due to a stronger and more resilient EMS [52]. However, the influence of pandemic is neither consistent with the objective of our study, nor decisive for evaluating post-OHCA intra-hospital mortality risk in relation to patient’s characteristics and level of care of first admission hospital. In addition, it is essential to acknowledge that the inclusion criterion of survival beyond 24 h post-hospital admission, while methodologically justifiable and consistent with prior OHCA studies, could introduce inherent selection bias. This criterion excluded patients who did not survive the initial 24 h, potentially impacting the generalizability of our findings. In conclusion, an acknowledged limitation of this study is the absence of data on functional recovery after OHCA; while Cerebral Performance Category (CPC) scores are available for survivors within our Registry, this information is not uniformly accessible for all patients.

Our study has also important strengths. Lombardia CARe is the most complete multicentre longitudinal prospective OHCA registry nationwide, with automated and accurate data-gathering processes. The longitudinal perspective adopted by the study allows for a thorough examination of trends and variations in post-OHCA intra-hospital mortality risk, and the multicentre approach ensures the inclusion of diverse geographic and demographic factors. In addition, the adoption of the 2014 Utstein standardized criteria ensures reliability and consistent reporting of OHCA cases. Moreover, study population has been selected among more than 12 thousand records, collected on a very diverse territory and characterised by different morphologies and various levels of urbanisation, over a period of 8 years. These peculiarities make the results and consequent reflections generalisable to many other areas and contexts.

In conclusion, our study shows that the intra-hospital mortality risk was 27% higher among OHCA patients with non-shockable presenting rhythm, net of the hospital level of care. This result has great implications in OHCA management and strategic planning. If multiple interventions in highly specialised centres do not improve the outcome for those patients with certain characteristics, the resources might be better allocated elsewhere. As for a public health approach, another important finding is that first CPR is performed by bystanders in almost 40% of cases, an element that, despite being positive in itself, leaves room for improvement. Since early defibrillation of shockable rhythms is associated with improved chances of survival, it is necessary to ensure timely access to defibrillators and implement education and training programs to the first CPR. A network of volunteer first responders trained in CPR and AED use, alerted by a short-text-message (SMS) system or by smartphone-based applications (APP) and dispatched to the OHCA site, has been implemented in other countries [53, 54]. It is the right path to follow, as also strongly recommended by international resuscitation and cardiological scientific societies [36].

## Data Availability

The raw data supporting the conclusions of this article will be made available by the authors, without undue reservation.
